# Artificial intelligence and cardiac surgery during COVID‐19 era

**DOI:** 10.1111/jocs.15417

**Published:** 2021-02-10

**Authors:** Raveena K. Khalsa, Arwa Khashkhusha, Sara Zaidi, Amer Harky, Mohamad Bashir

**Affiliations:** ^1^ St. George's Medical School, University of London London UK; ^2^ School of Medicine, Faculty of Health and Life Science University of Liverpool Liverpool UK; ^3^ School of Medicine King's College London London UK; ^4^ Department of Cardio‐thoracic Surgery Liverpool Heart and Chest Hospital Liverpool UK; ^5^ Vascular & Endovascular Surgery Royal Blackburn Hospital Blackburn UK

**Keywords:** big data, coronavirus, deep learning, imaging, telemedicine

## Abstract

The coronavirus disease 2019 (COVID‐19) pandemic has increased the burden on hospital staff world‐wide. Through the redistribution of scarce resources to these high‐priority cases, the cardiac sector has fallen behind. In efforts to reduce transmission, reduction in direct patient–physician contact has led to a backlog of cardiac cases. However, this accumulation of postponed or cancelled nonurgent cardiac care seems to be resolvable with the assistance of technology. From telemedicine to artificial intelligence (AI), technology has transformed healthcare systems nationwide. Telemedicine enables patient monitoring from a distance, while AI unveils a whole new realm of possibilities in clinical practice, examples include: traditional systems replacement with more efficient and accurate processing machines; automation of clerical process; and triage assistance through risk predictions. These possibilities are driven by deep and machine learning. The two subsets of AI are explored and limitations regarding “big data” are discussed. The aims of this review are to explore AI: the advancements in methodology; current integration in cardiac surgery or other clinical scenarios; and potential future roles, which are innately nearing as the COVID‐19 era urges alternative approaches for care.

## 1 INTRODUCTION

The announcement of the coronavirus disease 2019 (COVID‐19) pandemic by WHO, early in 2020, added significant burden to nations and healthcare systems.^1^, ^2^, ^3^ As a precaution for the surge in COVID‐19 patients, health instituitions across the globe have directed priority toward intensive care units.

The redistribution of limited resources such as ventilators, beds, healthcare staff, and PPE has precipitated an inadequate supply for cardiac surgery procedures.^2^, ^4^ Consequently, alike many other specialities, cardiac surgery has been affected significantly through: cancellation of elective cases; suspension of nonurgent cases; changes in the mode of preoperative assessment; and alterations in the follow‐up post discharge ‐ with the aim to protect both the hospital staff and the public. Arguably delays in treatment for some cardiac patients may pose risks, thus it is mandatory to find alternative forms of delivering the cardiac service to monitor cardiac patients whilst minimizing the risk of COVID‐19 transmission, as COVID‐19 is associated with higher morbidity and mortality in patients with existing cardiovascular diseases.^5^, ^6^ Henceforth, where possible, human contact should be avoided when addressing cardiac patient care to prevent the risk of critical COVID‐19 infected pneumonia.^7^

Subsequently, this led to issues with treatment, decision making and risk management, all of which drive the search for alternative decision‐making approaches that minimize contact between individuals, such as artificial intelligence (AI). AI is an umbrella term describing the ability of technology to process decisions, learn independently, and reflect on any mistakes. Advantages revolve around the empowerment to users by overriding traditional systems with faster accurate processing.^8^ Although developed from the computer science field, this technology provides an insight to procedures that can be integrated into the clinical framework during this pandemic and the future thereafter.^9^ Radiology has witnessed tremendous strides with the advent of ultrasound, computed tomography, magnetic resonance imaging, and positron emission tomography scanning technologies. The next breakthrough is AI utilizing the imaging data available from these imaging technologies.^10^ In particular to cardiac surgery, AI automation of analytical aspects such as time‐consuming clerical functions is deemed to be optimal for all. Through engulfment of laborious note keeping via computerization during patient visits, AI liberates physicians for more time promoting a patient‐centered holistic approach. Furthermore development of evidence‐based algorithms provides more accurate differential diagnoses, which is imperative in a multidisciplinary approach that lacks coherence in the current era.^11^ The aim of this literature review is to summarize AI: its current advancement; COVID‐19 influences on its development; and its potential future role in cardiac surgery.

## 2 DEEP LEARNING, MACHINE LEARNING, AND BIG DATA

**Figure 1:**
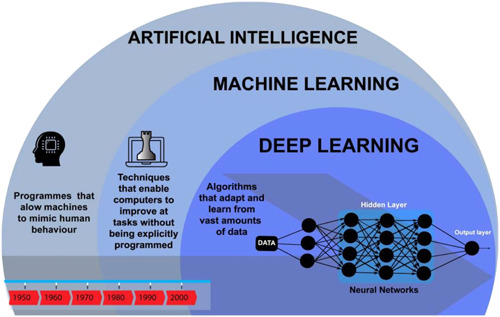
Evolution of artificial intelligence from 1950 to 2000^12^

Two subsets of AI are machine learning (ML) and deep learning (DL) (Figure 1). Machine Learning is the study of a specific computer algorithms that is built through a mathematical algorithm model from sample data which in turn is used to generate predictions or decisions. DL is far more complex than ML and this involves the utilization of artificial neural networks with representation learning. It can also be thought as a form to automate the predictive analytics and therefore DL is linked to a hierarchy of increasing complexity and abstraction while ML algorithms remain a linear approach.

DL is superior to ML due to differences in analysis of data. In ML, data is input into a model, which labels said data, identifies features then generate an output. For example, photos of carotid vessels can be input, labeled as carotid, specific features then identified (i.e.carotid atheromatous plaques) to finally output symptomatic or asymptomatic classification based on the images.^13^ DL although similar utilizes a neural network further complicating the input/output system, this branch of ML can be loosely described as an artificial manifestation of the brain. The neurons in the mind are mimicked by nodes (Figure 1). One row of nodes is linked to a subset of concealed nodes; this hierarchal network is triggered by the former first layer of nodes, leading to a cascade of activation. Alike neurones, these nodes require an “all‐or‐nothing action potential” to be activated. Once these “all‐or‐nothing” inputs reach the final layer of nodes, the data has been inferred in a nonlinear manner. The advantage of nonlinear interpretation is being able to identify more complex features, improving the interpretation.^14^ The need for AI in healthcare is growing in this pandemic due to the accumulation of patient data and the requirement to assess each area of data, from history to images. These large volumes of data, termed “big data” are collected by big data platforms.^15^

## 3 TECHNOLOGY AND CARDIAC SURGERY

**Figure 2:**
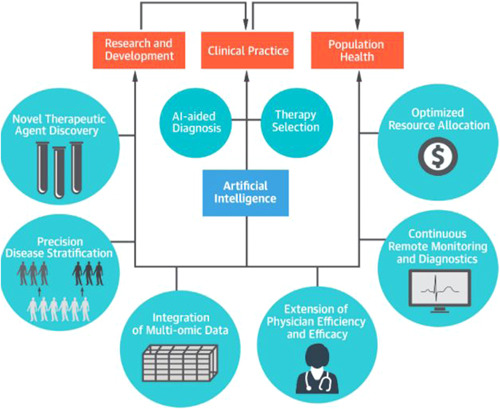
Different areas where artificial intelligence can be used in cardiovascular medicine^14^

Telemedicine has seen an exponential growth during COVID‐19 era as direct patient contact is mitigated. Many functions of telemedicine identified include: monitoring chronic conditions, rehabilitation, specialist consultations, real‐time assessment of clinical status, and more.^16^ A meta‐analysis showed tele‐cardiac‐rehabilitation was significantly associated with reduced hospitalizations and cardiac events (relative risk = 0.56, 95% confidence interval [CI] = 0.39–0.81, *p* < .001) compared with usual care.^17^ Despite these benefits, limitations include lack of internet, smart devices, technology knowledge, patient confidentiality concerns, and inadequate alternative for a full physical examination. However, the application of tele‐medicine has paved a path for AI.^18^ AI has potential roles in aiding stages of cardiac surgery, such as scanning, diagnosing, risk assessment, and treatment plans^3^ (Figure 2). One of the most popular neural networks in the literature are convolutional neural networks (CNN). Although deep learning was initially used for analyzing computer images, an early medical use of CNN was shown in a study comparing the classification by ophthalmologists versus AI to identify diabetic retinopathy. The gold standard was determined by several physicians, in comparison AI required a large collection of images (128,175 images), highlighting one of the downfalls of AI. Within cardiology, CNNs have been applied to computer‐aided technology such as electrocardiograms to learn to identify any features (i.e.abnormalities). The performance in classifying ventricular and supraventricular ectopic beats exceeded “state‐of‐the‐art methods.” As the system was not overtly specific, its use applies to any electrocardiogram data set.^19^ In comparison, a study by Narula et al.^20^ identifies features of heart “tissue deformation” to differentiate between left ventricular hypertrophy versus hypertrophic cardiomyopathy (HCM). To identify HCM, “speckle‐tracking echocardiographic data” was utilized from 77 athletes. Despite the machine model obtaining higher sensitivity and specificity than the doppler‐derived transmitral velocities, a cohort of athletes is not representative for the performance of DL on the general population.^20^

A clinical study conducted by Saeyeldin et al.^21^ demonstrated AI aiding triage for ascending thoracic aortic aneurysms (ATAAs). To assess whether patients required surgery an algorithm was constructed based on aortic size; more than or equal to 5 cm, prophylactic surgery was recommended.^21^ Similarly, Ruiz‐Fernández et al.^22^ evaluated the use of AI, independent of the gold standard classification (Risk Adjustment for Congenital Heart Surgery) to differentiate risks (of mortality and other complications) of congenital heart surgery. It was found that AI‐based algorithms for decision support have the potential to assess patients' risk who undergo congenital heart surgery into low, medium, and high complexity cases. Using multilayer perceptron self‐organizing maps, radial basis function networks and decision trees, these algorithms were seen to have 80%–99% accuracy. Eventhough these four different algorithms were utilized, the “multilayer perceptron” algorithm achieved the highest accuracy (range, 81.79%–99.87%). Considering an algorithm produces 18.21% inaccurate results, selection of algorithms needs to be carefully evaluated to reduce errors, but the study does suggest the algorithms are useful predictors of mortality in cardiac surgery and can allow surgeons to prepare more appropriately and effectively for the procedure.^22^

Both Saeyeldin et al.^21^ and Ruiz‐Fernández et al.^22^ highlight the same message that information from AI about risks allows anticipation of treatment plans, overall significantly contributing to clinical decisions. Jalali at el.^23^ also found that ML algorithms and wavelet analysis can be accurately applied to prediction models to assess the occurrence of periventricular leukomalacia in neonates postcardiac surgery. Artificial neural networks (ANNs) nonlinear technology are equally useful in identifying risk factors and predicting mortality in patients who have undergone cardiac surgery. In a prospective study of 18,362 cardiac surgery patients, out of 72 total variables, ANNs were able to selectively rank 34 of the most relevant predictors for mortality, yielding an area under the receiver operating curve (AUROC) of 0.82 (95% CI: 0.80–0.83). The operative mortality in patients undergoing coronary artery bypass surgery using the ANN model, had an AUROC of 0.80 (0.77–0.82) compared with 0.78 (0.75–0.80) using the logistic EuroSCORE. Following the same trend, in patients undergoing a valve procedure, the AUROC was 0.76 (0.73–0.79), which is higher than with the logistic EuroSCORE 0.72 (0.69–0.75). Therefore, this highlights the greater discriminatory power of AI systems in prediction models compared with traditional assessment tools.^24^ Finally, lifestyle, genetics, gut microbiome sequencing and diet are important factors, which can influence a patient's state of health and disease thus, in turn, influencing electronic health records and big data. The ability to process big data into tools to allow for cognitive computing, DL and ML enable the unique potential for AI to drive precision cardiovascular medicine.^25^

## 4 THE DRAWBACKS

Nevertheless, there are many drawbacks to AI largely concerning patient data. In order to achieve an ideal machine learning model, copious amounts of data is required. A reason explaining the gap between numerous advancements in AI but it's slow implantation into cardiac surgery can be accounted to the need for data. Data availability, governance, ownership, quality, standardization and user‐friendliness are a few criterions revolving big data platforms. Standardization of data input is also tedious as healthcare systems conduct varied formats of data collection.^15^ Although data is now more widely available and accessible than ever, the quality of that data determines the success of the model.^26^ The model is only aware of situations which are presented through the data set, which must be able to eliminate bias towards any one factor. Once presented with new unfamiliar data, the models often tend to malfunction; a major limitation within imaging as not all patient cases are identical, thus the questionable reliability and biases of these algorithms may reduce confidence.^27^

## 5 SCEPTICS

The birth of AI has cultivated different attitudes from adept learners to analytical sceptics. The mechanism behind AI although described previously still withholds an area of complexity not fully understood. The understanding of AI can be divided into the white, gray and black‐box, where the colors represent the transparency of the method. One issue to many is the “black‐box problem”, this conundrum revolves around the opacity of complex AI such as neural networks. As AI is explored and researched further, more explainable AI (XAI) may calm the suspicions from sceptics. But this study comes at a cost and not all believe pinpointing the processes will aid the development of AI; either way efforts still need to be made by all users to continue learning as AI continues to flourish.^28^ Parties opposing AI also voice concerns around the lack of human interaction as AI invades into numerous specialties and trust issues surrounding the predictions made by algorithms.^11^ However, supervised Smart Tissue Autonomous Robot surgery has successfully displayed suturing of the bowel and “real‐time feedback” provided by AI has aided pressure adjustment during surgical practice on soft tissues.^29^ Over time, presentation of such examples, may be the only way to minimize concerns and encourage the idea of AI alongside physicians rather than replacement as the introduction of AI into the clinical framework is merely the beginning of the future human–machine companionship.

## 6 THE FUTURE WITH AI

A future implementing AI within cardiology and other practices is promising, but COVID‐19 has mounted more pressure to accelerate this physician–computer partnership. For example, both the volume and interpretation of patient data is overwhelming and AI offers efficient assistance in analysis of all aspects of patient data, from symptoms to imaging and more. Secondly, algorithms calculating predictions of mortality risk, other complications and disease severity is a powerful contribution to determining the best patient treatment and prognosis. Finally, the large number of mundane tasks, such as documenting consultations, that physicians are responsible for may be replaced by AI speech recognition, freeing more time for patient care and ideally more patients to be seen within the same time period; a welcomed concept whilst staff are outstretched during the pandemic.^11^

## 7 CONCLUSION

AI has paved its path into the healthcare system and with the growing numbers of data more efficient alternatives to aid the clinical framework are embraced. Considering the pandemic, the more AI alternatives available reduce the likelihood of COVID‐19 transmission and the burden on limited resources. However, aforementioned challenges surrounding data should not be underestimated and require time to resolve.

## CONFLICT OF INTERESTS

All the authors declare that there are no conflict of interests.

## References

[1] Cascella M , Rajnik M , Cuomo A , Dulebohn SC , Di Napoli R . Features, Evaluation, and Treatment of Coronavirus. In: StatPearls [Internet]. In: StatPearls [Internet]. Treasure Island (FL): StatPearls Publishing. 2020. PMID: 32150360.

[2] Kermali M , Khalsa RK , Pillai K , Ismail Z , Harky A . The role of biomarkers in diagnosis of COVID‐19: a systematic review. Life Sci. 2020;254:117788. 32475810 10.1016/j.lfs.2020.117788 PMC7219356

[3] Suri JS , Puvvula A , Biswas M , et al. COVID‐19 pathways for brain and heart injury in comorbidity patients: a role of medical imaging and artificial intelligence‐based COVID severity classification: a review. Comput Biol Med. 2020;124:103960. 32919186 10.1016/j.compbiomed.2020.103960 PMC7426723

[4] Ad N , Luc JGY , Nguyen TC , et al. Cardiac surgery in North America and coronavirus disease 2019 (COVID‐19): regional variability in burden and impact. J Thorac Cardiovasc Surg. 2020. 10.1016/j.jtcvs.2020.06.077 PMC7330597 32768300

[5] Haft JW , Atluri P , Ailawadi G , et al. Adult cardiac surgery during the COVID‐19 pandemic: a tiered patient triage guidance statement. Ann Thorac Surg. 2020;110(2):697‐700. 32305286 10.1016/j.athoracsur.2020.04.003 PMC7161520

[6] Huang L , Zhao P , Tang D , et al. Cardiac involvement in recovered COVID‐19 patients identified by magnetic resonance imaging. JACC Cardiovasc Imaging. 2020;13(11):2330‐2339. 10.1016/j.jcmg.2020.05.004 32763118 PMC7214335

[7] Wang D , Hu B , Hu C , et al. Clinical characteristics of 138 hospitalized patients with 2019 novel coronavirus‐infected pneumonia in Wuhan, China. JAMA ‐ J Am Med Assoc. 2020;323(11):1061‐1069. 10.1001/jama.2020.1585 PMC7042881 32031570

[8] Dilsizian SE , Siegel EL . Artificial intelligence in medicine and cardiac imaging: harnessing big data and advanced computing to provide personalized medical diagnosis and treatment. Curr Cardiol Rep. 2014;16(1):1‐8. https://link.springer.com/article/10.1007/s11886-013-0441-8 10.1007/s11886-013-0441-8 24338557

[9] Shi F , Wang J , Shi J , et al. Review of artificial intelligence techniques in imaging data acquisition, segmentation and diagnosis for COVID‐19. IEEE Rev Biomed Eng. 2020;14:4‐15. 10.1109/RBME.2020.2987975 32305937

[10] King BF . Artificial intelligence and radiology: what will the future hold? J Am Coll Radiol. 2018;15:501‐503. https://pubmed.ncbi.nlm.nih.gov/29371088/ 29371088 10.1016/j.jacr.2017.11.017

[11] Bashir M , Harky A . Artificial intelligence in aortic surgery: the rise of the machine. Semin Thorac Cardiovasc Surg. Vol 31, 2019:635‐637. 31279913 10.1053/j.semtcvs.2019.05.040

[12] De Marvao A , Dawes TJW , Howard JP , O'Regan DP . Artificial intelligence and the cardiologist: What you need to know for 2020. Heart. 2020;106:399‐400. http://heart.bmj.com/ 31974212 10.1136/heartjnl-2019-316033 PMC7035692

[13] Stoitsis J , Valavanis I , Mougiakakou SG , Golemati S , Nikita A , Nikita KS . Computer aided diagnosis based on medical image processing and artificial intelligence methods. Nucl Instrum Methods Phys Res Sect A Accel Spectrometers, Detect Assoc Equip. 2006;569(2 SPEC. ISS):591‐595.

[14] Johnson KW , Torres Soto J , Glicksberg BS , et al. Artificial Intelligence in Cardiology. J Am Coll Cardiol. 2018;71:2668‐2679. http://creativecommons.org/licenses/by/4.0/ 29880128 10.1016/j.jacc.2018.03.521

[15] Raghupathi W , Raghupathi V . Big data analytics in healthcare: promise and potential. Heal Inf Sci Syst. 2014;2(1):1‐10. http://www.hissjournal.com/content/2/1/3 10.1186/2047-2501-2-3 PMC4341817 25825667

[16] Flodgren G , Rachas A , Farmer AJ , Inzitari M , Shepperd S . Interactive telemedicine: effects on professional practice and health care outcomes. Cochrane Database Syst Rev. 2015;(9):CD002098. 26343551 10.1002/14651858.CD002098.pub2 PMC6473731

[17] Thomas E , Gallagher R , Grace SL . Future‐proofing cardiac rehabilitation: transitioning services to telehealth during COVID‐19. Eur J Prev Cardiol. 2020. http://journals.sagepub.com/doi/10.1177/2047487320922926 10.1177/2047487320922926 PMC7928989 33611474

[18] Adam S , Zahra SA , Chor CYT , Khare Y , Harky A . COVID‐19 pandemic and its impact on service provision: a cardiology prospect. Acta Cardiol. 2020:1‐8. https://www.tandfonline.com/doi/full/10.1080/00015385.2020.1787636 10.1080/00015385.2020.1787636 32646309

[19] Kiranyaz S , Ince T , Gabbouj M . Real‐time patient‐specific ECG classification by 1‐D convolutional neural networks. IEEE Trans Biomed Eng. 2016;63(3):664‐675. 26285054 10.1109/TBME.2015.2468589

[20] Narula S , Shameer K , Salem Omar AM , Dudley JT , Sengupta PP . Machine‐learning algorithms to automate morphological and functional assessments in 2D echocardiography. J Am Coll Cardiol. 2016;68(21):2287‐2295. 27884247 10.1016/j.jacc.2016.08.062

[21] Saeyeldin A , Zafar MA , Li Y , et al. Decision‐making algorithm for ascending aortic aneurysm: effectiveness in clinical application? Thorac Cardiovasc Surg. 2019;157(5):1733‐1745. 10.1016/j.jtcvs.2018.09.124 30579535

[22] Ruiz‐Fernández D , Monsalve Torra A , Soriano‐Payá A , Marín‐Alonso O , Triana Palencia E . Aid decision algorithms to estimate the risk in congenital heart surgery. Comput Methods Programs Biomed. 2016;126:118‐127. https://pubmed.ncbi.nlm.nih.gov/26774238/ 26774238 10.1016/j.cmpb.2015.12.021

[23] Jalali A , Simpao AF , Gálvez JA , Licht DJ , Nataraj C . Prediction of periventricular leukomalacia in neonates after cardiac surgery using machine learning algorithms. J Med Syst. 2018;42(10):1‐11. https://link.springer.com/article/10.1007/s10916-018-1029-z 10.1007/s10916-018-1029-z PMC12400904 30116905

[24] Nilsson J , Ohlsson M , Thulin L , Höglund P , Nashef SAM , Brandt J . Surgery for Acquired Cardiovascular Disease Risk factor identification and mortality prediction in cardiac surgery using artificial neural networks. J Thorac Cardiovasc Surg. 2006;132:12‐21. 16798296 10.1016/j.jtcvs.2005.12.055

[25] Krittanawong C , Zhang HJ , Wang Z , Aydar M , Kitai T . Artificial Intelligence in Precision Cardiovascular Medicine. J Am Coll CardiolElsevier USA. 2017;69:2657‐2664. 10.1016/j.jacc.2017.03.571 28545640

[26] Vlahogianni EI . Artificial Intelligence Applications to Critical Transportation Issues [cited 2020. Sep 9]. Available from. http://onlinepubs.trb.org/onlinepubs/circulars/ec168.pdf

[27] Naudé W . Artificial intelligence vs COVID‐19: limitations, constraints and pitfalls. AI Soc [Internet]. 2020;35:761‐765. 10.1007/s00146-020-00978-0 PMC7186767 32346223

[28] Adadi A , Berrada M . Peeking inside the black‐box: a survey on explainable artificial intelligence (XAI). IEEE Access. 2018;6:52138‐52160.

[29] Rimmer L , Howard C , Picca L , Bashir M . The automaton as a surgeon: the future of artificial intelligence in emergency and general surgery. Eur J Trauma Emerg Surg, 2020:1‐6. https://link.springer.com/article/10.1007/s00068-020-01444-8 10.1007/s00068-020-01444-8 32715331

